# Ape Conservation Physiology: Fecal Glucocorticoid Responses in Wild *Pongo pygmaeus morio* following Human Visitation

**DOI:** 10.1371/journal.pone.0033357

**Published:** 2012-03-15

**Authors:** Michael P. Muehlenbein, Marc Ancrenaz, Rosman Sakong, Laurentius Ambu, Sean Prall, Grace Fuller, Mary Ann Raghanti

**Affiliations:** 1 Department of Anthropology, Indiana University, Bloomington, Indiana, United States of America; 2 Sabah Wildlife Department, Kota Kinabalu, Sabah, Malaysia; 3 Kinabatangan Orangutan Conservation Programme, Sandakan, Sabah, Malaysia; 4 North of England Zoological Society, Chester, United Kingdom; 5 Red Ape Encounters, Sukau, Sabah, Malaysia; 6 Cleveland Metroparks Zoo, Cleveland, Ohio, United States of America; 7 Department of Biology, Case Western Reserve University, Cleveland, Ohio, United States of America; 8 Department of Anthropology, School of Biomedical Sciences, Kent State University, Kent, Ohio, United States of America; Texas A&M University, United States of America

## Abstract

Nature-based tourism can generate important revenue to support conservation of biodiversity. However, constant exposure to tourists and subsequent chronic activation of stress responses can produce pathological effects, including impaired cognition, growth, reproduction, and immunity in the same animals we are interested in protecting. Utilizing fecal samples (N = 53) from 2 wild habituated orangutans (*Pongo pygmaeus morio*) (in addition to 26 fecal samples from 4 wild unhabituated orangutans) in the Lower Kinabatangan Wildlife Sanctuary of Sabah, Malaysian Borneo, we predicted that i) fecal glucocorticoid metabolite concentrations would be elevated on the day after tourist visitation (indicative of normal stress response to exposure to tourists on the previous day) compared to samples taken before or during tourist visitation in wild, habituated orangutans, and ii) that samples collected from habituated animals would have lower fecal glucocorticoid metabolites than unhabituated animals not used for tourism. Among the habituated animals used for tourism, fecal glucocorticoid metabolite levels were significantly elevated in samples collected the day after tourist visitation (indicative of elevated cortisol production on the previous day during tourist visitation). Fecal glucocorticoid metabolite levels were also lower in the habituated animals compared to their age-matched unhabituated counterparts. We conclude that the habituated animals used for this singular ecotourism project are *not* chronically stressed, unlike other species/populations with documented permanent alterations in stress responses. Animal temperament, species, the presence of coping/escape mechanisms, social confounders, and variation in amount of tourism may explain differences among previous experiments. Acute alterations in glucocorticoid measures in wildlife exposed to tourism must be interpreted conservatively. While permanently altered stress responses can be detrimental, preliminary results in these wild habituated orangutans suggest that low levels of predictable disturbance can likely result in low physiological impact on these animals.

## Introduction

Tourism generates more than 9% of the global gross domestic product [1], and may account for almost half of the GDP in developing countries with biodiversity-rich ecotourism draws [2]. Whale- and dolphin-watching activities in 1998 alone generated more than US$1 billion [3]. Ecotourism represents “environmentally responsible travel and visitation to relatively undisturbed natural areas, in order to enjoy and appreciate nature (and any accompanying cultural features — both past and present) that promotes conservation, has low visitor impact, and provides for beneficially active socio-economic involvement of local populations”, p. 20 [4]. Ecotourism accounts for a significant proportion of all international tourism and contributes billions of dollars to the national income of various countries [5]. Such revenue could enhance economic opportunities for local residents, support environmental education, and protect the natural and cultural heritage of the area, including the conservation of biodiversity and improvement of local facilities [6]–[8].

Rapid, unmonitored development of nature-based tourism projects can also lead to degradation of habitats and deleterious effects on animal well-being, negatively impacting the very species we wish to conserve [9]. Animals can become crowded in restricted habitats, pollution may increase in otherwise pristine areas, and invasive species could be introduced accidentally. Animals can become displaced, altering their activity budgets and preventing them from feeding properly [10]–[15]. Animals used for tourism may also have reduced opportunities to interact with unhabituated animals not used for tourism, prohibiting social interactions. Habituation of animals to human presence can increase the likelihood that animals will actively seek out contact with humans, particularly in the form of crop raiding and invasion of garbage pits and latrines. Habituation may make animals more vulnerable to poaching because of their loss of fear of humans.

Tourists also likely serve as a source of infection, particularly to nonhuman primates who are genetically closely related to humans and share a number of common infections. A significant proportion of visitors to primate rehabilitation centers are not adequately vaccinated and are likely infectious, creating unnecessary risk of pathogen transmission to wildlife because they are largely unaware of the impacts they may directly have on animal health, despite their interests in environmental protection [16]–[18].

Anthropogenic disturbances may also affect adversely animal physiology. Stressors are noxious stimuli that trigger responses to maintain homeostasis. Normal activation of stress responses (both sympathetic nervous system and hypothalamic-pituitary adrenal axis) is important for survival via alteration of metabolism and behaviors. Chronic exposure to stressors can lead to ‘allostatic overload’ [19]. However, constant exposure to stressors and chronic activation of stress responses can produce pathological effects, including impaired cognition, growth, reproduction, and immunity [20]–[25]. The chronic stressor of tourism exposure to wildlife could theoretically cause immunosuppression, increasing susceptibility to infectious diseases, and decreasing reproductive success.

Field endocrinology has recently been used to assess alterations of the hypothalamic-pituitary-adrenal axis in endangered and economically valuable wildlife species [26]–[32]. Fecal samples collected noninvasively under natural conditions are the ideal media for assessing extended secretion of glucocorticoid metabolites in response to anthropogenic stressors in wildlife [33]. Fecal extracts represent the accumulation of glucocorticoids and their metabolites over a period of time approximately equal to gut retention time of the animal. Because of this, and because concentrations of glucocorticoid metabolites in feces parallel concentrations of glucocorticoids in plasma [34], fecal glucocorticoid metabolites (fGM) have been used to assess stress in a variety of animal species [35]–[37]. For example, in areas with elevated recreational intensity (compared with animals in other areas), higher fGM levels were identified in wolves (*Canis lupus*), pine martens (*Martes martes*), elk (*Cervus elaphus*), and wood grouse (*Capercaillie Tetrao urogallus*) [35], [38]–[40].

The present study proposed to evaluate the effects of tourist exposure on fGM levels of wild habituated orangutans. Fecal steroids [41]–[46] have been used previously to describe endocrine correlates of behavior [47], [48], reproduction [49], [50], and immune function [51], among other things, in populations of wild nonhuman primates. Here we aimed to test the null hypothesis that wild orangutans exposed to tourists as part of the Red Ape Encounters (RAE) program in Sabah, Malaysian Borneo, exhibit normal fGM responses, indicative of a lack of chronic stress in these animals. According to the Red List of Threatened Species, Bornean orangutans (*Pongo pygmaeus* ssp.) are endangered [52]. Orangutan tourism has focused mainly around rehabilitation centers that use tourism for public education and to generate resources for conservation activities [53]. Red Ape Encounters (see below) is the only ecotourism program that facilitates trekking for wild orangutans. Given the increasing demand of tourists to encounter wild orangutans, it is critical to evaluate any potential physiological effects this and future programs may have on this charismatic and endangered species. We predicted that: 1) fGM concentrations would be elevated on the day after tourist visitation (indicative of normal stress response to exposure to tourists on the previous day) compared to samples taken before or during tourist visitation; 2) samples collected from unhabituated animals not used for tourism would have higher fGM concentrations than from those habituated animals utilized for tourism.

## Materials and Methods

### Ethics statement

Ethical approvals were obtained from the Institutional Animal Care and Use Committee of Indiana University (protocol #08-047) and by the Scientific Research Review Committee of Cleveland Metroparks Zoo (project #CS2005-030). Permission to conduct this research was granted by the Sabah Wildlife Department. As fecal samples were collected noninvasively from the captive and wild orangutan populations, all research complied with all recommendations of the 2006 Weatherall report for the use of non-human primates in research.

### Samples for fecal degradation test

Under field conditions, it is not usually possible to process fecal samples immediately following defecation or collection. Therefore, we determined the effects of time on hormone degradation using samples from one male and one female adult orangutan (*Pongo pygmaeus pygmaeus*) at Cleveland Metroparks Zoo. Using a wooden applicator stick, first-morning samples were collected between 7 and 8 am (immediately following defecation) off of a clean enclosure floor, uncontaminated with water or urine. Three samples were collected from each animal on non-consecutive days. Immediately following defecation, a portion of the fecal mass was stored in a closed centrifuge tube at enclosure temperature. A portion of each sample was removed from the tube and processed (see below) precisely every 30 minutes for up to 6 hours (for a total of 78 measurements {39 measurements per animal}; 6 fGM values for each of 13 different 30-minute time points). Solid-phase extraction for this portion of the project took place in a laboratory at Cleveland Metroparks Zoo; sample solvolysis and enzyme immunoassay took place in the Evolutionary Physiology and Ecology Laboratory at Indiana University.

Because gut biota, diet and environmental conditions differ between captive animals at Cleveland and wild animals in Borneo, we also collected a single fecal sample from each of two previously unknown, unhabituated wild orangutans (one adult male and one adult female) when they were encountered during area surveys in the Kinabatangan (see below). Like the Cleveland samples, these samples (presumably first morning voids before 8 am) were collected immediately following defecation and processed in a similar fashion described above (a solid-phase extraction every 30 minutes for up to 6 hours) so as to determine the effects of time on hormone degradation.

### Samples for tourist visitation test

**Figure 1:**
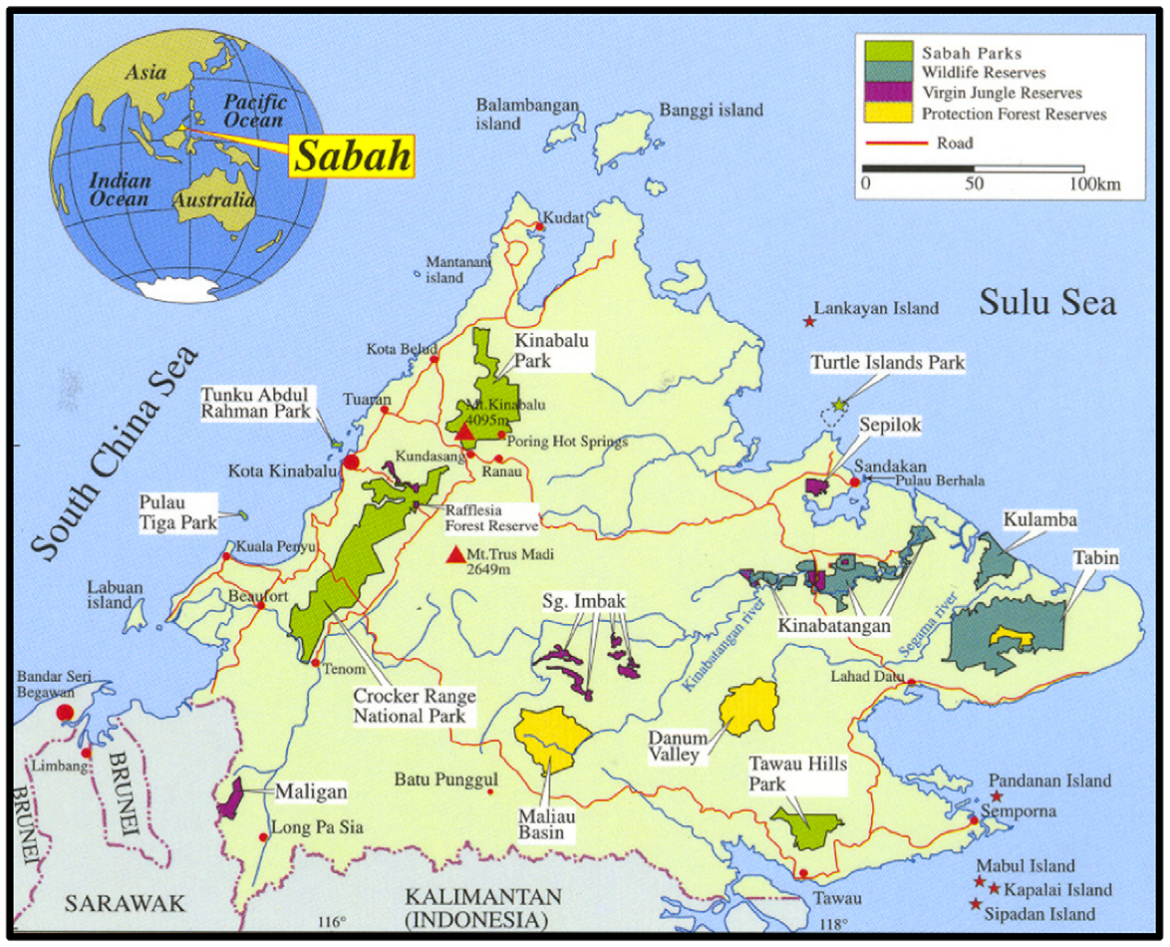
Location of Sabah, Malaysian Borneo. Illustration provided by the Sabah Wildlife Department.

**Figure 2:**
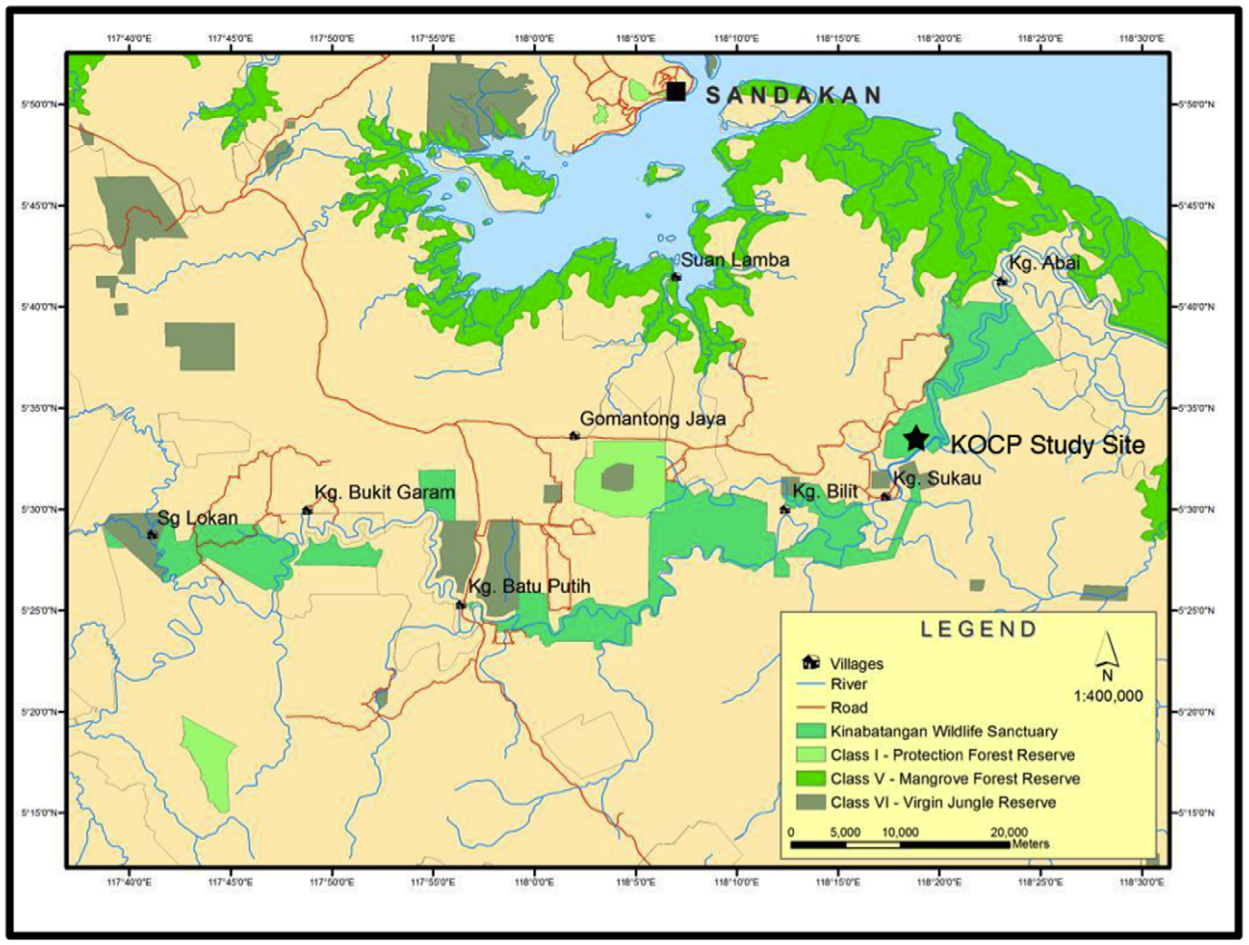
Location of Kinabatangan Orangutan Conservation Programme (KOCP) study site where Red Ape Encounters bring tourists to visit wild orangutans.

Between 2008 and 2009, 36 fecal samples were collected and processed from Jenny, an adult (at the time, approximately 32 years of age) female orangutan (*Pongo pygmaeus morio*), and 17 samples from her 11-year old sub-adult male offspring, Etin. These wild animals have been monitored semi-continuously since 1998 as part of the Kinabatangan Orangutan Conservation Programme (KOCP) near the village of Sukau, in the Malaysian state of Sabah (north Borneo) (Figures 1 and 2). These animals inhabit the 26,000 hectare Lower Kinabatangan Wildlife Sanctuary, an area of refuge surrounded by encroaching agricultural productions. The lowland dipterocarp forest is home to approximately 1,100 orangutans (of the approximately 55,000 left in Borneo), 8 other species of primates, Bornean elephants, estuarine crocodiles, more than 300 species of birds, and other wildlife [54]. KOCP has been involved in general bio-monitoring, particularly of orangutan population trends, analysis of orangutan behavioral ecology, and community-oriented initiatives in Sabah.

In 2001, KOCP, the Sabah Wildlife Department and the Danish International Development Agency formed the Red Ape Encounters (RAE) community-based ecotourism program in the village of Sukau in the Lower Kinabatangan Wildlife Sanctuary, an area acknowledged as a prominent tourism destination in Sabah's National Ecotourism Plan [55]. Of the tourism operations in the Kinabatangan, RAE is the only community-owned and operated program (with significant socioeconomic return to indigenous people of Sukau and for conservation purposes), and the only agency that facilitates trekking for wild orangutans [54], [56]. RAE is therefore one of few true ecotourism programs in Borneo. Their program is designed to enhance knowledge about the local wildlife and environment, clearly communicating conservation messages. Visitor guidelines are similar to those described in the Best Practice Guidelines for Wild Great Ape Tourism by the International Union for Conservation of Nature (IUCN) [57]. For example, sick individuals are not allowed to participate, a maximum of 7 tourists are allowed with the animals at any given time, length of visitation with the animals is limited to 1 hour (with animals rarely exposed to tourists for more than 3 hours per week), distance between visitors and orangutans is limited to 10 meters minimum, and visitors must adopt appropriate behaviors at all times [58]. Currently, RAE hosts approximately 250 tourists per year, with most of the activity centered around two habituated orangutans: Jenny and Etin. This is the only commercial operation that regularly brings tourists to view wild orangutans.

To evaluate changes in fecal cortisol levels associated with tourist visitations, our goal was to obtain fecal samples from both Jenny and Etin i) 24 hours before tourist visitation (to act as a baseline, with no human exposure within the preceding 48 hours), ii) during tourist visitation (to measure the effects of the previous day's exposure to our two field assistants) and iii) 24 hours after tourist visitation (to measure the effects of the previous day's exposure to the tourists). But obtaining samples for each animal and time point is difficult because of the relatively few tourists that presently utilize RAE, and because these animals are completely wild and utilize a very large territory, coming and going as they please. It is very difficult to stay with a single animal for more than a few days at a time, leading to a compromised sampling schedule.

Samples were collected in association with 25 tourist visitation events. Of the 53 samples (36 from Jenny and 17 from Etin), 17 were obtained on the day before tourist visitation, 24 were obtained during tourist visitation, and 12 were obtained the day after tourist visitation. Samples were collected by 8 am each day (which may represent first morning void when the animals first awoke from their night nest) to minimize any diurnal effects. Animal identification and behaviors (including potential behavioral indicators of stress like hair pulling, self-scratching, vocalizations, hiding, fleeing, teeth baring, and defensive head turns), animal physical condition, height of animal in canopy, tourist behaviors (including grouping, volume of conversation, camera flashes, etc.), number of tourists present, distance between animal and tourists, date, time of animal defecation and sample extraction were recorded. Given the nature of fecal hormone secretion, elevated fGM levels detected in the samples obtained the day after tourist visitation would be indicative of elevated stress response on the previous day (during the tourist visitation), assuming a gut retention time of approximately 24 hours for a large-bodied primate with a high fibrous diet [48], [59], [60]. The average gut retention time of wild orangutans is unknown.

Fecal samples were also obtained from 4 previously unidentified (and unhabituated) orangutans (2 sub-adult males and 2 adult females) when they were encountered during area surveys, expeditions and line transects in the Kinabatangan. Once these animals were located, they were followed every day that was possible. Ten fecal samples were obtained from the 2 unknown females (9 samples collected upon initial encounter, or an encounter with no previous contact for at least 2 days; only 1 sample was collected within a day after previous encounter), and 16 samples from the 2 unknown males (10 samples collected upon first encounter, or an encounter with no previous contact for at least 2 days; and 6 samples collected within a day after previous encounter).

Solid-phase extraction for this portion of the project took place in the KOCP laboratory in Sukau; sample solvolysis and enzyme immunoassay took place in the Evolutionary Physiology and Ecology Laboratory at Indiana University.

### Solid-phase extraction

Each sample was thoroughly mixed within its collection tube. 0.2 grams (g) of feces (no fibrous plant or other undigested material) was weighed and mixed with 2.5 milliliters (ml) of distilled water and 2.5 ml of 95% ethanol in a new tube. The tube was then vortexed for exactly 5 minutes (min) (it was critical that each sample be processed exactly the same way to ensure equal extraction). The tube was then centrifuged at 3300 revolutions per minute (rpm) for 10 min, and the fecal extract (supernatant) decanted into a clean tube. An Alltech Prevail C18 Maxi-Clean reversed-phase non-polar silica solid-phase extraction cartridge (500 milligrams [mg] bed weight) was primed with 2 ml of distilled water. Using a syringe, 2 ml of the fecal extract was slowly loaded into the cartridge, followed by another 2 ml of distilled water. Both ends of the cartridge were capped, the cartridge was labeled and placed into a zip-lock bag containing a silica gel packet. Cartridges were stored at ambient temperature in the dark for two weeks before being shipped to the laboratory and frozen at −20 degrees Celsius (°C). This method of adhering the fecal analytes onto a sorbet matrix in the field requires the availability of organic solvents and electricity, but also allows for shipping diagnostic, non-infectious samples at ambient temperature, eliminating the need for shipping alcohol, dry ice or liquid nitrogen. This method was also preferred in the present study because of unpredictable freezer/electricity availability in the forest.

Using a vacuum manifold, the cartridges were washed using 1 ml of 20% methanol and eluted using 2 ml of 100% methanol. The eluted samples were evaporated to dryness using an Organomation Multi-Vap nitrogen evaporator (70°C, 15 pounds per square inch [PSI]). Samples were rehydrated with 1 ml of 100% ethanol and vortexed. Samples underwent solvolysis (50 microliters [µl] of a sulfuric acid solution [6.94 ml H_2_SO_4_ with 43.06 ml water], 100 µl of a saturated sodium chloride solution [31.13 g made up to 100 ml water], and 4.5 ml of ethyl acetate, mixed with sample, incubated overnight in a water bath [40°C]) to hydrolyze conjugated steroids. The next morning, 2.5 ml of water were added, the tubes vortexed for 5 min, and then centrifuged for 3 min at 1000 rpm. The ethyl acetate fraction was transferred into clean tubes, evaporated until dryness and rehydrated with 1 ml of 100% ethanol.

### Enzyme immunoassay

Cortisol antibody and cortisol-horseradish peroxidase (HRP) were obtained from Coralie Munro at the University of California-Davis. 200 µl of antibody were mixed with 1.8 ml carbonate buffer (2 Sigma carbonate-bicarbonate capsules with 200 ml water). 124 µl of this solution were mixed with 198 ml of carbonate buffer to yield a 1∶16,000 antibody dilution. 100 µl of this was pipetted into each well of a Nunc Maxisorb plate. The plates were incubated inside a humidity chamber in a refrigerator overnight, after which they were washed (4 cycles with 10 second soaks on a Bio-Tek ELx-405 plate washer) with buffer (1800 ml water with 200 ml concentrated buffer [87.66 g NaCl, 5 ml Tween 20, made up to 1000 ml water]), blotted, 150 µl buffer (10.842 g NaH_2_ PO_4_.H_2_O, 17.324 g Na_2_ HPO_4_, 17.4 g NaCl, 2 g bovine serum albumin, made up to 2000 ml water; pH adjusted to 7) added to each well, sealed and frozen until use.

Eight standard concentrations (5–1000 picograms [pg] per well) of cortisol were created by dilution of a stock solution (10 mg of cortisol in 100 ml 100% ethanol). 900 µl of unknowns were transferred into clean tubes, and these along with standard and controls (62.5 pg and 17 pg) were evaporated to dryness. 300 µl of cortisol-HRP conjugate solution (17 ml buffer with 11.3 µl HRP solution [5 µl HRP with 495 µl buffer]) were added to each tube, vortexed, and then transferred to a deep-well plate. 200 µl of the cortisol-HRP conjugate solution were added to the zero concentration well, and 300 µl buffer to the non-specific binding well.

Antibody-coated plates were removed from the freezer and allowed to reach room temperature within a humidity chamber, after which the buffer was dumped and the plates blotted. 100 µl of from each well in the deep-well plate were then pipetted in duplicate to the corresponding wells on the antibody-coated plate. The plate was sealed and incubated for 2 hours (5 min on a plate rotator and then 155 min in the humidity chapter at room temperature). The plate was washed with buffer as before, and 100 µl of substrate-citrate buffer solution (250 µl of 2,2′-azino-bis[3-ethylbenzothiazoline-6-sulphonic acid] [ABTS]-solution [0.439 g ABTS made up to 20 ml water] with 80 µl H_2_O_2_ solution [0.5 ml H_2_O_2_ with 7.5 ml water], and 24.67 ml citrate buffer [19.21 g citric acid with 1966 ml water, pH adjusted to 4]) were added to each well. The plate was resealed and rotated for 5 min, and incubated in the humidity chamber for 25 min at room temperature. 100 µl stop solution (10 ml of water with 20 µl ethylenediaminetetraacetic acid (EDTA) solution [41.623 mg EDTA made up to 100 ml water] and 10 ml hydrofluoric acid solution [5.048 ml of 49% hydrofluoric acid with 1.2 NaOH [5 normal]) were added to each well. Absorbance (optical density) of each well was read at 415 nanometers (nm) (reference wavelength of 570 nm) using a Bio-Tek Synergy 2 plate reader. The average optical density of the non-specific binding wells was subtracted from the average optical density of all other wells. Percent binding was plotted against the standard concentrations using a log-logit transformation. Based on the dilutions used above, the value of each unknown was transformed from pg per well to nanograms (ng) of glucocorticoid metabolite per gram of feces by multiplying by 0.042, with separate dilution factors for standards and controls. A detailed protocol can be made available upon request. This assay was adapted from previous methods used by Dr. Toni Ziegler and Dan Wittwer at the Wisconsin National Primate Research Center for other nonhuman primate species [61], [62].

### Statistical analyses

Data were entered into an Excel database that was then imported into SAS version 9.1 (SAS Institute, Cary, NC) for statistical analysis. Intra-assay coefficients of variation were assessed using the mean coefficients of variation of control duplicates. Intra-assay coefficients of variation were less than 8%. Interassay coefficients of variation were assessed using the mean coefficients of variation of control (high and low) duplicates in five separate assays. Interassay coefficients of variation were less than 10%.

Continuous measures were summarized by mean, median, and standard deviation. For the fecal degradation samples (N = 2 Cleveland {3 samples per Cleveland animal} and 2 wild animals), average cortisol values for each time point (every 30 minutes between 0 and 6 hours) were plotted, and a first-order auto correlation was used to evaluate change over time. For the Cleveland samples, a repeated measures analysis of variance was run on their sequential samples (3 samples collected on non-consecutive days) to evaluate stability in fGM across days.

Samples from the wild habituated animals (Jenny and Etin) collected before (N = 17), during (N = 24) and after (N = 12) tourist visitation were compared using analysis of co-variance (to control for amount of time between defecation and extraction, although this window was always less than 3 hours). Samples from the 4 unhabituated animals collected at initial encounter (N = 19) and after exposure to researchers (N = 7) were compared using analysis of co-variance. Samples from the wild, habituated animals were compared with those of the unhabituated animals (at corresponding time point: before visitation for Jenny and Etin versus initial encounters for the unhabituated animals, and after visitation for Jenny and Etin versus secondary encounters for the unhabituated animals) as well as the captive Cleveland samples using non-parametric Mann-Whitney U tests. Level of significance (alpha) was set at 0.05. Because of low sample size (and subsequent power less than 80%), borderline significant results are interpreted conservatively to avoid conclusions based on false negatives.

## Results

### Fecal degradation

**Figure 3:**
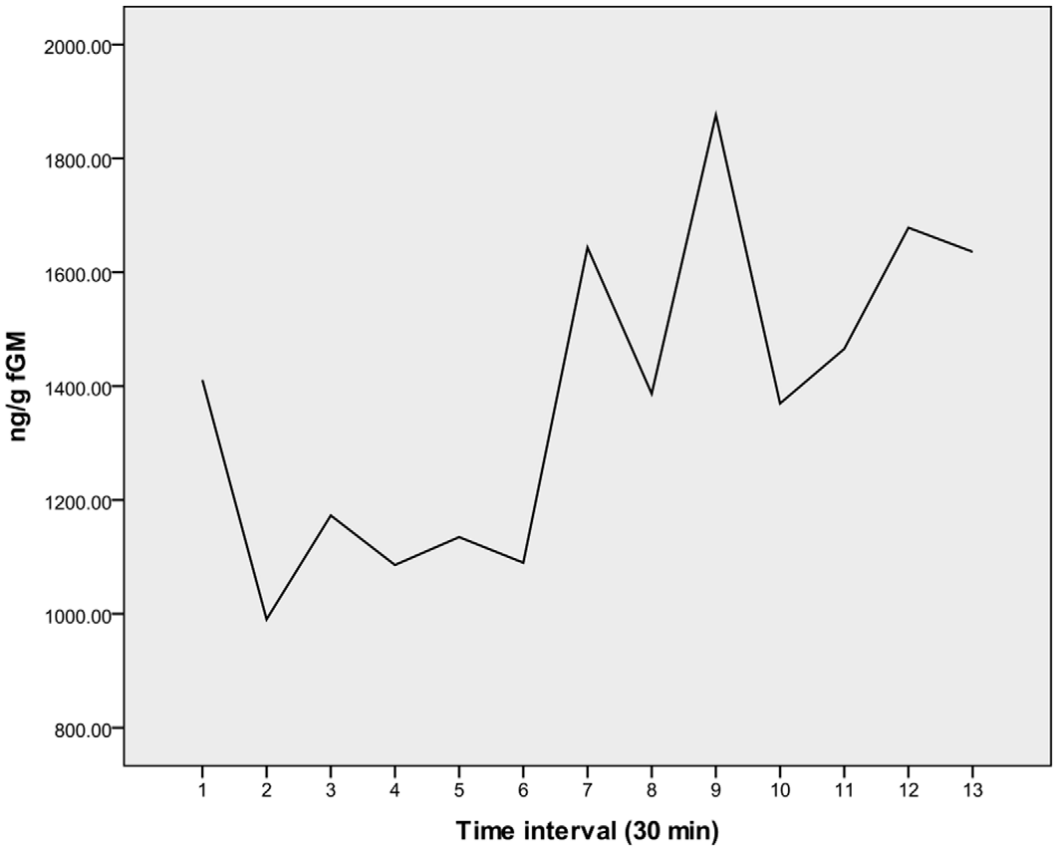
Analysis of fecal glucocorticoid metabolite degradation over time in captive and wild animals (4 animals total, 8 measurements at each time point) indicates that, if samples cannot be extracted immediately (time point 1), they should be extracted between 1 and 3 hours of defecation (between time points 2 and 6 on figure). The time between defecation and sample extraction should also be recorded.

Under field conditions, it is not usually possible to process fecal samples immediately following defecation or collection. Therefore, we determined the effects of time on hormone degradation using samples from one male and one female adult orangutan (*Pongo pygmaeus pygmaeus*) at Cleveland Metroparks Zoo as well as two wild orangutans (*Pongo pygmaeus morio*) in Borneo. Results suggest that, if samples cannot be extracted immediately following defecation, then they should be extracted within 3 hours following defecation. At that point, variation in fGM increases substantially (Figure 3). Results of the repeated measures analysis on the Cleveland samples indicate stability in fGM within individuals across days (within-subjects effects, F = 1.657, p = 0.141), but with increased variability within days if samples are processed after more than three hours.

Increased variation in fGM over time likely reflects bacterial metabolism, conjugate cleavage and other physiological processes [63]–[65]. With this knowledge, *we always processed fecal samples from the wild orangutans within three hours of defecation* (mean time between defecation and extraction of 2 hours). We contend that results from other studies dependent upon samples collected randomly without witnessing defecation, and those not extracted (or frozen) within 3 hours of defecation, are limited. The time between defecation and extraction should be standardized for a given research population, ideally with all samples and cohorts extracted at approximately the same time since defecation. Samples extracted immediately can yield very different results from those extracted after 3 hours.

### Effects of tourist visitation and researcher contact

Of the 25 different tourist visitations that were monitored, mean number of tourists at each visitation was 6.2 (SD 5.1) with a mean length of visit of 143 min (SD 111). These variables were unassociated with fGM levels in either animal (results not shown). The two habituated animals exhibited no behavioral indicators of stress (i.e., no kiss-squeak or agonistic vocalizations, hiding or fleeing, or branch breaking) and animal physical condition appeared excellent. Height of animal in canopy, tourist behaviors (including grouping, volume of conversation, camera flashes, etc.), number of tourists present, and distance between animals and tourists did not vary significantly to allow further analysis.

In comparing Jenny and Etin's (the habituated animals') samples obtained the day before, the day of and the day after tourist visitation (ANCOVA controlling for time between defecation and sample extraction), fGM levels were significantly elevated in samples collected the day after tourist visitation (indicative of elevated cortisol production on the previous day: during tourist visitation) (F = 3.140, p = 0.052).

For the unknown animals, fGM levels in samples obtained after previous contact with researchers were numerically higher but not statistically significantly different from their initial samples (F = 0.070, p = 0.794; ANCOVA again controlling for time between defecation and sample extraction) (see Table 1).

**Table 1:** 

Orangutans	Sample paradigm§	Mean ng/g fGM (SD)
Habituated (N = 2)	Before tourists (N = 17)	1272 (526)
	During tourists (N = 24)	1367 (704)
	After tourists (N = 12)	1933 (1336)
Unhabituated (N = 4)	First contacts (N = 19)†	1821 (812)
	Secondary contacts (N = 7)‡	2188 (1630)

§ Indicates number of samples collected for each time period.

† Samples collected upon initial contact with unhabituated animals as well as samples collected from these same animals after no previous contact with researchers for at least 2 days.

‡ Samples collected within 24 hours of previous contact with researchers.

### Comparisons between habituated and unhabituated animals

In comparing the habituated animals' (Jenny and Etin's) samples obtained the day before tourist visitation (N = 17; their baseline samples reflective of no contact with humans the day prior) with the samples obtained from the 2 unhabituated animals upon initial contact or no contact with humans in the 2 preceding days (N = 19), fGM levels were lower in the habituated animals (z = −2.234, p = 0.025). However, fGM levels in the habituated animals' samples the day after tourist visitation (N = 12; when fGM levels are elevated in response to tourist exposure on the previous day) were not statistically different (z = −0.169, p = 0.902) from samples obtained from the 2 unhabituated animals the day after exposure to the researchers (N = 7).

## Discussion

fGM levels following an anthropogenic stressor provide valuable biometric data that complement previous work illustrating behavioral alterations in wild populations of nonhuman primates in response to habituation and tourism [66]–[68]. For example, mountain gorillas used in tourism spend more time moving and monitoring and exhibit more self-directed indicators of stress (e.g., self-grooming and scratching) compared to other gorillas [69]. Other studies indicate costs of primate-based tourism, like increased food-associated aggression in adult Tibetan macaques with subsequent major increases in infant mortality [70], increased frequency of threat displays in Tibetan macaques in response to tourist noises [71], and worse coat condition in ring-tailed lemur groups [72]. Populations of black howlers (*Alouatta pigra*) in Belize regularly visited by tourists have significantly higher fGM values than other study groups not visited by tourists [73]. fGM levels were elevated in Barbary macaques (*Macaca sylvanus*) in Morocco only following ‘aggressive’ human behavior towards the animals (and not other tourist behaviors), although self-scratching in males (used here as a proxy for anxiety) was related directly to rates of all tourist-macaque interactions [74].

The present study, although not without limitations, utilizes a more powerful study design (sampling the same animals before, during and after tourist exposure, across several tourist visitation events, in the absence of potential social or nutritional stressors) to identify apparently normal fGM responses to tourist exposure in wild orangutans, with fGM levels increasing in response to tourist visitation, and then returning to a lower level. fGM levels were also lower in habituated than in nonhabituated animals. We therefore conclude that these habituated animals utilized for tourism in Sabah are not chronically stressed, unlike other animals with documented permanent alterations in stress responses. For example, habituated wild adult Magellanic penguins (*Spheniscus magellanicus*) in Argentina exposed to tourism exhibit blunted corticosterone responses following capture and restraint as well as blunted responses following exogenous treatment with adrenocorticotropin hormone [75], [76]. They also exhibit lowered baseline glucocorticoid concentrations compared to non-tourist-visited animals [77]. In contrast, Australian Little penguins (*Eudyptula minor*) at tourist sites exhibit higher glucocorticoid concentrations than animals at non-tourist sites [78], and tourist-exposed Yellow-eyed penguins (*Megadyptes antipodes*) exhibit significantly higher corticosterone responses to capture and restraint [79]. Tourist-exposed marine iguanas (*Amblyrhynchus cristatus*) in the Galápagos islands exhibit reduced stress responses to capture and restraint compared to more isolated animals [80]. Others have identified elevated corticosterone concentrations in iguanas at different tourist sites, with elevated stress responses to restraint and handling compared to animals at undisturbed sides [81]. Others have identified no effects of tourism on glucocorticoid responses in Pyrenean chamois (*Rupicapra pyrenaica pyrenaica*) [82] or spotted hyenas (*Crocuta crocuta*) [83]. It would appear that different species and populations of animals react differently to human exposure. Differences may also be due, in large part, to variation in tourism intensity (including distance between animals and visitors) and stage of habituation.

It is difficult to interpret the differential causes and consequences of hypo- versus hyper-secretion of glucocorticoids. Insight might be found in cases the etiology of post-traumatic stress disorder (PTSD). Those with PTSD (including civilian trauma like loss of loved one, disaster, sexual abuse, etc.) typically display lower baseline (tonic) cortisol levels compared to healthy controls [84]–[88]. In contrast, PTSD patients show elevated reactive (phasic) cortisol levels when asked to review personalized trauma scripts [89]. The cause of lowered baseline levels of cortisol in PTSD may be a combination of altered function of the adrenal cortex [90] and enhanced negative feedback on the hypothalamus and anterior pituitary [91].

Other studies have paradoxically observed increased baseline cortisol levels in those with PTSD [92], [93], suggesting that hypo- and hyper-secretion are both evident in PTSD. This might be explained by variation in stressor type and personality factors. For example, “uncontrollable” stressors (like abuse, disaster or loss) are frequently associated with chronically elevated cortisol levels [94]. Furthermore, while humans habituating to stress (e.g., Trier Social Stress Test) sometimes exhibit lower salivary and plasma cortisol over time [95], this habituation likely happens most readily in only sub-groups of participants (‘low responders’) with specific personality traits (those with more self-esteem, less physical health problems and less depressed mood) [96].

Similarly, animal temperament may influence the outcome of anthropogenic disturbance, with some animals responding more negatively to human disturbance [97], [98]. In fact, animals likely disperse based partly on individual temperaments as well as the distribution of food, mates and predators. Therefore “endocrinal differences between animals occupying disturbed and undisturbed areas may not be solely a direct effect of stress response to disturbance by humans, but may also reflect the non-random spatial distribution of individuals of different temperaments”, p. 66–67 [98]. In short, elevated fGM levels in tourist-exposed versus unexposed groups [73], as well as elevated fGM in animals interacting (like aggressively, or during feeding) versus not interacting (or neutral interactions) with tourists [74] must be interpreted more conservatively. The effects of stress on wildlife populations are likely context dependent. Elevated fGM may simply reflect normal response to stimuli, with little effects on fitness in the presence of coping/escape mechanisms [99]. Furthermore, it is possible that previous studies are confounded by social interactions among animals (i.e., dominance and sexual interactions among animals resulting in elevated fGM) as well as variations in diet between animals/groups.

Tourism seems to negatively affect fitness of some wildlife populations. Survival is lower in tourist-exposed groups of Amazonian hoatzin juveniles (*Opisthocomus hoatzin*) than undisturbed groups [100]. Similarly, Yellow-eyed penguins (*Megadyptes antipodes*) exposed to tourism exhibit significantly lower breeding success than those in other populations [79]. Permanently altered acute stress responses in habituated animals could be detrimental because normally-functioning acute responses are necessary for ‘fight-or-flight’ reactions [23], [25]. Such costs would be higher in pregnant females for which elevated glucocorticoids would be problematic for both the female and her fetus [101]–[103].

Whatever the case may be, nature-based tourism programs that result in permanent alterations of stress physiology in their animals should not be viewed as ‘sustainable,’ since they violate the basic ‘do no harm’ principle of ecotourism. With increasing demand from tourists to experience direct encounters with wildlife, it is important to produce definitive guidelines that will protect wildlife. Funds must be redirected directly from these programs into species conservation, enabling the employment of more local community members to supervise tourist-animal interactions. Third-party monitoring programs (formed from organizations such as the International Union for the Conservation of Nature, International Ecotourism Society, Convention on Biological Diversity, Conservation International, the Cooperative Research Centre for Sustainable Tourism, the World Tourism Organization and the United Nations Environment Programme) could be utilized to ensure the balance between animal well-being and economic opportunities. Furthermore, animal usage must be kept minimal (at least for pregnant females and ill animals, and during times of resource restriction), and the habituation processes gradual. The costs associated with tourism development must be carefully considered before new groups of animals are habituated. And most importantly, consumer behavior and visitor management must change. The public must be informed better about the risks they may pose to wildlife.

Low sample sizes (for reasons explained above) prevent us from concluding if acute variation in cortisol in these orangutans correlates with survival or reproduction probabilities. It is unknown if there are changes in neurotransmitter or endogenous opioid levels or receptor functions in these animals. An adrenocorticotropic hormone (ACTH)-challenge as well as a ‘capture-and-restraint’ test would certainly yield useful information [104], but are not feasible with wild orangutans. The present sample also cannot assess diurnal cortisol patterns or the cortisol awakening response, both of which can be informative for chronic stress [105]–[108]. However, the orangutans at this site are generally in excellent health (no visible external signs of illness) with no predation, few social stressors (these animals are largely solitary) and little seasonal fluctuation in food resources. Low levels of predictable disturbance likely result in low physiological impact on these animals. This may be particularly the case with tempered (‘low responding’) animals with adequate coping or escape mechanisms. Wildlife at this and other sites should be continually monitored for potential adverse reactions, and the results of continued monitoring of fGM must be interpreted conservatively (i.e., that acute elevation in fGM represents normal response to stimuli, particularly in social species) in the absence of fitness data or physiological measures indicative of chronic stress. In the interim, we continue to recommend relatively low levels of tourism (i.e., predictable, heavily regulated disturbance) of wild apes, although best practices must be location/population/species-specific [57].
